# Membrane Damage and Metabolic Disruption as the Mechanisms of Linalool against *Pseudomonas fragi*: An Amino Acid Metabolomics Study

**DOI:** 10.3390/foods13162501

**Published:** 2024-08-09

**Authors:** Jiaxin Cai, Haiming Chen, Runqiu Wang, Qiuping Zhong, Weijun Chen, Ming Zhang, Rongrong He, Wenxue Chen

**Affiliations:** 1 HSF/LWL Collaborative Innovation Laboratory, College of Food Sciences & Engineering, Hainan University, 58 People Road, Haikou 570228, China; cjx19990905@163.com (J.C.); hmchen168@126.com (H.C.); hainufood88@163.com (Q.Z.); chenwj@hainu.edu.cn (W.C.); zhangming-1223@163.com (M.Z.); rongronghe@hainanu.edu.cn (R.H.); 2 Department of Biostatistics, University of Nebraska Medical Center, Omaha, NE 68198, USA; ruwang@unmc.edu

**Keywords:** linalool, *Pseudomonas fragi*, antibacterial mechanism, membrane damage, metabolism disruption

## Abstract

*Pseudomonas fragi* (*P. fragi*) is usually detected in low-temperature meat products, and seriously threatens food safety and human health. Therefore, the study investigated the antibacterial mechanism of linalool against *P. fragi* from membrane damage and metabolic disruption. Results from field-emission transmission electron microscopy (FETEM) and atomic force microscopy (AFM) showed that linalool damage membrane integrity increases surface shrinkage and roughness. According to Fourier transform infrared (FTIR) spectra results, the components in the membrane underwent significant changes, including nucleic acid leakage, carbohydrate production, protein denaturation and modification, and fatty acid content reduction. The data obtained from amino acid metabolomics indicated that linalool caused excessive synthesis and metabolism of specific amino acids, particularly tryptophan metabolism and arginine biosynthesis. The reduced activities of glucose 6-phosphate dehydrogenase (G6PDH), malate dehydrogenase (MDH), and phosphofructokinase (PFK) suggested that linalool impair the respiratory chain and energy metabolism. Meanwhile, genes encoding the above enzymes were differentially expressed, with *pfkB* overexpression and *zwf* and *mqo* downregulation. Furthermore, molecular docking revealed that linalool can interact with the amino acid residues of G6DPH, MDH and PFK through hydrogen bonds. Therefore, it is hypothesized that the mechanism of linalool against *P. fragi* may involve cell membrane damage (structure and morphology), disturbance of energy metabolism (TCA cycle, EMP and HMP pathway) and amino acid metabolism (cysteine, glutamic acid and citrulline). These findings contribute to the development of linalool as a promising antibacterial agent in response to the food security challenge.

## 1. Introduction

Foodborne diseases should be taken seriously worldwide because of their considerable incidence rate and mortality [1,2]. Foods can be contaminated with pathogenic organisms from harvest to consumption and ultimately lead to foodborne illnesses including colitis, uremia, typhoid fever and acute gastroenteritis [3]. Cold meat accounts for a large proportion in people’s daily diet because of its high nutritional value. However, the environment of cold meat is particularly suitable for the growth of *Pseudomonas* spp. due to its having higher water activity and lower temperature. Among the *Pseudomonas* spp., *Pseudomonas fragi* (*P. fragi*) is one of the most threatening species to cold meat spoilage, which can lead to a decrease in its quality [4]. *P. fragi*, as a rod-shaped psychrophile, has the ability to adapt to low temperature and break down proteins and fats [5]. Studies have shown that *P. fragi* contains a large number of genes related to amino acid transport metabolism, suggesting that the bacteria may be more involved in amino acid transport metabolism [6]. When *P. fragi* grows on meat, it secretes extracellular proteases that cause structural changes and odor. The emergence of odor is caused by the production of volatile malodorous compounds, such as esters, amines and aldehydes, when amino acids are broken down [7]. Therefore, it is imperative to find an effective method to reduce spoilage caused by Pseudomonas berries. Current methods of killing foodborne pathogens in food mostly use physical and chemical methods, while common physical sterilization affects the product quality and nutrient loss. In addition, consumers may be concerned about resistance and potential toxicological issues associated with the use of synthetic preservatives. Therefore, there is an urgent need to find an effective method to reduce food spoilage caused by *P. fragi*.

Plant essential oils (EOs), as natural preservatives, are receiving more and more attention due to their safety and degradability. As a secondary metabolite of plants, EOs are generally considered safe (GRAS) and contain a large number of bioactive compounds with antibacterial, antioxidant, and anti-inflammatory functions [8]. EOs consist of a wide range of components in varying concentrations, of which those with antimicrobial properties are mainly phenolic acids, terpenes, aldehydes and flavonoids. Linalool (3,7-dimethyl-1,6-octadien-3-ol) is the main antibacterial component isolated from plants, such as cinnamon, lavender, and citrus essential oils [9]. As a natural monoterpene compound, linalool is a colorless transparent liquid that can volatilize with a special aroma [10]. Linalool is soluble in organic solvents such as propylene glycol because of its polar hydrocarbon structure [11]. Linalool has been shown to have a wide range of biologically active properties, such as reducing inflammation, treating neuralgia, and possessing anticancer effects [12,13,14]. It also has excellent antibacterial properties; the minimum inhibitory concentration (MIC) of linalool against *P. fragi* was 1.5 mL/L, which was superior to 3-carene and *Myrtus communis*, as mentioned in other studies [15,16]. It has also been previously found that the MICs of linalool against common *Campylobacter* spp. and *Shigella flexneri* were 1 and 4 mL/L, respectively [17,18]. The discrepancies between our results and other reports regarding MIC values can be ascribed to the different chemical profiles of coriander essential oils, different experimental protocols and different strains used in studies [19]. For example, Gram-positive and -negative bacteria vary in cell wall structure, composition, acid resistance and drug sensitivity. The antibacterial effect of linalool may be attributed to its structure, in which the hydrophobic group exhibits interactions with the hydrophobic peptidoglycan and membrane core of bacteria [20]. By disrupting the lipid structure of the cell membrane, the permeability of the cell membrane is altered. As a result, cell metabolism is blocked and thus inactivated [21]. A previous report also confirmed that linalool treatment can induce the production of intracellular reactive oxygen species (ROS) of *Shigella sonnei* and increase membrane lipid oxidation [22]. Furthermore, studies have indicated that linalool can enhance the preservation of chilled beef by inhibiting *Brochothrix thermosphacta* and *Pseudomonas lundensis* [23,24]. Studies have analyzed the antibacterial mechanism of *P. fragi* by omics, including transcriptomics and metabolomics [25,26]. However, the underlying mechanism of linalool against *P. fragi* with regard to membrane damage and amino acid metabolomics is still unclear. Therefore, in this study, targeted metabolomics was employed to research the amino acid metabolism of *P. fragi*. In contrast to the previous studies, which investigated the mechanism of bacterial inhibition only at the cellular and metabolic levels, the present study also explored the molecular level.

Advanced microscopy methods have both high-resolution imaging and ultra-sensitive capabilities [27]. Therefore, tools such as atomic force microscopy (AFM), field-emission transmission electron microscopy (FETEM) and confocal laser scanning microscopy (CLSM) have been introduced in this experiment for in-depth study of cellular morphological changes. In this study, the morphology and membrane integrity of *P. fragi* were observed using AFM, CLSM and FETEM. Fourier transform infrared (FTIR) was also performed to detect changes in the chemical composition of the cell membrane structure. Enzymes related to respiratory energy metabolism and targeted amino acid metabolism were also investigated in order to further reveal the potential mechanisms at the metabolic level. Then, qRT-PCR and molecular docking simulation were performed to progressively resolve the modes of action of linalool at the molecular level. The results provide a valuable theoretical basis for controlling *P. fragi* during low-temperature meat product processing and storage.

## 2. Materials and Methods

### 2.1. Materials and Reagents

*P. fragi* (CGMCC1.3349) was obtained from the China General Microorganism Culture Collection and Management Center. The strain was activated with nutrient agar (NA) (Beijing Land Bridge Technology Co., Ltd., Beijing, China) and incubated at 30 °C. Linalool (purity > 99%) was provided by Hainan Camphora Biotech Co., Ltd. (Haikou, China). Glucose 6-phosphate dehydrogenase (G6PDH), malate dehydrogenase (MDH), and phosphofructokinase (PFK) kits were procured from Suzhou Grace Biotechnology Co., Ltd. (Suzhou, China). The Bacterial Genome DNA Extraction Kit was purchased from Beijing Solarbio Science & Technology Co., Ltd. (Beijing, China). All other chemicals and reagents were of analytical grade unless otherwise specified.

### 2.2. Investigation of Cell Morphology

#### 2.2.1. AFM Observation

The morphological changes in bacterial cells were observed using AFM (Bruker Multimode 8-HR, MA, USA) according to a previous report [28]. The MIC of linalool used in this study was 1.5 mL/L based on previous research [25]. In brief, *P. fragi* was cultured overnight, and the bacterial suspension was collected using centrifugation (6000× *g*, 10 min, 4 °C) at 4 h after adding linalool. Samples without linalool served as a control. Subsequently, the suspensions were fixed with polyformaldehyde at 4 °C for 15 min, centrifuged (6000× *g*, 10 min, 4 °C) and washed with sterile distilled water. The samples were dropped on a freshly peeled mica sheet and dried. Finally, the samples were subjected to AFM with a probe (SCANASYST-AIR) under a scan area of 5.0 × 5.0 μm and frequency of 1.0 Hz.

#### 2.2.2. FETEM Observation

FETEM observation was performed in order to visualize the morphological changes in *P. fragi* [29]. The bacteria were cultured at 30 °C with shaking at 150 rpm for 12 h and then treated with linalool for 2 h, with sterile distilled water used as a control group. Subsequently, bacterial cells were collected using centrifugation at 6000× *g* for 10 min and then resuspended in phosphate-buffered saline (PBS) (0.1 mol/L, pH 7.4). The morphology of *P. fragi* was observed using FETEM (Thermoscientific Talos F200X G2, Waltham, MA, USA).

### 2.3. Cell Membrane Integrity

#### 2.3.1. CLSM Observation

The effects of linalool on cell membrane integrity in *P. fragi* were analyzed using a Live/Dead cell double staining kit (G1707) as described previously [30]. Briefly, bacteria were cultured to the logarithmic growth phase and subsequently harvested using centrifugation (6000× *g*, 10 min). The precipitate was resuspended in sterile saline (0.85% NaCl) prior to adding Calcein-AM and propyl iodide (PI). Subsequently, the mixture was incubated in the dark for 15 min. Finally, the sample was dropped on a glass slide with CLSM (Olympus, FV3000, Tokyo, Japan) to obtain fluorescence images.

#### 2.3.2. FTIR Spectroscopy

The chemical contents of the cell surface were extracted to assess changes in the chemical structure of cell membranes using FTIR spectroscopy as described in [31]. The samples were cultured overnight and then treated with linalool for 4 h. The bacterial suspensions were centrifuged and washed three times with PBS and then freeze-dried to harvest the samples. The spectra of the pellets (KBr:bacterial samples = 1:100, *w*/*w*) were observed at a wavelength range of 4000–400 cm^−1^.

### 2.4. Leakage of Intracellular Material

The effect of linalool on intracellular soluble proteins of *P. fragi* was investigated using SDS-PAGE as previously described [32]. *P. fragi* was incubated to the logarithmic growth phase and then washed three times with PBS (6000× *g*, 10 min) before being resuspended in the MIC of linalool. The samples were taken out for sonication (300 W, pause for 5 s, work for 5 min) on ice at 4, 8 and 12 h, respectively. Subsequently, the sample was mixed with an equal amount of loading buffer, heated at 100 °C for 5 min to denature the protein, and then cooled in an ice water bath. The protein bands were analyzed via SDS-PAGE with Coomassie Brilliant Blue R250 used as the staining solution.

### 2.5. Measurement of Intracellular Enzyme Activity

Enzyme activities related to respiration and energy production were measured in accordance with the method described by Sun et al. [33]. Briefly, activated *P. fragi* was cultured to the logarithmic growth phase and treated with the MIC of linalool for 0, 2, 4, 6 and 8 h, respectively. Meanwhile, nutrient broth containing test bacterial cells without linalool was set as the control group. Subsequently, the bacterial precipitate was collected with centrifugation (6000× *g*, 10 min, 4 °C) and resuspended in PBS for sonication (power: 300 W; sonicate for 3 s at 10 s intervals; repeat 30 times) to release intracellular enzymes. Enzyme activities including G6PDH, MDH and PFK were quantified following the manufacturer’s protocol.

### 2.6. Targeting Amino Acid Metabolomics Technology

The amino acid profiles were examined using ultra-high-performance liquid chromatography–mass spectrometry according to [34]. The samples were obtained as in Section 2.3.2 and then vortexed for 30 s and homogenized at 40 Hz for 240 s. The samples were sonicated in an ice-water bath for 5 min, and the above steps were repeated three times. After that, pre-cooled acetonitrile (−40 °C) and methanol mixtures (including isotope labelling internal standard mixture) were added and vortexed for 30 s to mix. Subsequently, the samples were allowed to stand 1 h after centrifuging (6000× *g*, 10 min, 4 °C). The supernatant was rotary evaporated to dryness and reconstituted with methanol and derivative agent (1-fluoro-2-4-dinitrophenyl-5-L-alanine amide). The solution was put into a water bath (40 °C) for 1 h and then rotary evaporated and reconstituted.

The UHPLC separation was carried out using a Vanquish UHPLC system (Thermo Fisher, MA, USA) equipped with a Waters ACQUITY UHPLC BEH C18 column (100 × 2.1 mm, 1.7 μm). Mobile phase A (ammonium acetate in water, 5 mM) and B (acetonitrile) were used to elute the samples in a column at 45 °C. The samples (2 μL) were injected onto the column with an auto-sampler at 4 °C and analyzed using Altis TSQ Plus mass spectrometry (Thermo Fisher, MA, USA) equipped with an electrospray ionization interface in multiple reaction monitoring (MRM) mode. Typical ion source parameters were as follows: spray voltage = −3300 V, sheath gas = 40 Arb, aux gas = 10 Arb, sweep gas = 1 Arb, ion transfer tube temperature = 325 °C, vaporizer temperature = 350 °C. Xcalibur 4.4.16.14 (Thermo Fisher Scientific Inc., Waltham, MA, USA) was employed for MRM data acquisition with data formatted using SIMCA16.0.2 (Sartorius Stedim Data Analytics AB, Umea, Sweden). The metabolic pathways were analyzed with KEGG (https://www.genome.jp/kegg/, accessed on 9 May 2023) and MetaboAnalyst (https://www.metaboanalyst.ca/, accessed on 10 May 2023).

### 2.7. Effects on DNA Expression

#### 2.7.1. Extraction of DNA

The bacteria were cultured overnight at 30 °C and then treated with/without linalool for 4 h. Next, the cells were harvested (8000× *g*, 10 min, 4 °C), washed three times and dissolved in PBS. The DNA of *P. fragi* was extracted using the Solarbio Bacterial Genome DNA Extraction Kit (D1600, Beijing Solarbio Science & Technology Co., Ltd., Beijing, China) according to the manufacturer’s instructions. Firstly, the collected cell precipitates were dissolved in solution A, and RNaseA was added for 5 min at room temperature. Subsequently, the protease was added to a water bath at 70 °C for 10 min, and then Solution B was added to continue the water bath. The solution was transferred to the adsorption column and mixed with anhydrous ethanol for centrifugation; then, rinsing solution was added, and the procedure was repeated three times. Finally, the DNA was collected by centrifugation with the addition of eluent and stored at −20 °C.

#### 2.7.2. Agarose Gel Electrophoresis

The agarose gel retarding experiments were performed to detect whether linalool binds to bacterial DNA and leads to inhibition or damage of DNA synthesis. The gel was prepared by mixing agarose with ethidium bromide (0.5 μg/mL) [35]. Agarose gel electrophoresis of DNA samples loaded with Tris-acetate-EDTA (TAE) running buffer was carried out with a constant voltage of 100 V for 30 min. Finally, the gel imaging system (UVsolo2 touch, Analytik Jena, Thuringia, Germany) was used to capture images.

#### 2.7.3. DEG Verification Using qRT-PCR

The genes encoding the above three enzymes (G6PDH, MDH and PFK) were selected from the previous transcriptomics of *P. fragi* for verification using qRT-PCR [26]. The cells of *P. fragi* were cultured overnight at 30 °C and then treated with the MIC of linalool for 4 h. The bacteria cells were centrifuged (8000× *g*, 10 min, 4 °C) and then resuspended in PBS. The total RNA of the *P. fragi* was obtained using an RNA extraction kit (Solarbio, Beijing, China) and subsequently quantified with a nanodrop spectrophotometer (Colibri Titertek Berthold, Germany). The isolated RNA was employed for cDNA synthesis by reverse transcription using a Fastking gDNA Dispelling RT SuperMix kit (Tiangen, Beijing, China). Finally, the cDNA samples were mixed with forward/reverse primers and a SuperReal PreMix Plus (SYBR Green) kit (Tiangen, Beijing, China) in a total volume of 20 µL. Premier 5 software was employed to design primers (Table 1) with the 16s RNA gene as a housekeeping gene. PCR was performed under the following reaction steps: pre-denaturation at 95 °C for 15 min, 40 cycles for denaturation at 95 °C for 10 s of annealing and extension at 65 °C for 30 s. The relative expression levels of the target genes and housekeeping genes were calculated according to the 2^−(ΔΔCt)^ method.

### 2.8. Molecular Docking

In this research, the binding mode and site of linalool with MDH, PFK, G6PDH were explored. The structure of linalool was obtained from ZINC (https://zinc.docking.org/, accessed on 24 June 2023), and 3D models of three enzymes were constructed with SWISS-MODEL (https://swissmodel.expasy.org/, accessed on 25 June 2023) using a previously outlined method [24]. Water molecules were deleted, and hydrogen atoms were added manually using Autodock Tools 4.2.7 [36,37]. The genetic algorithm was adopted to search for the best binding poses. After docking, 10 different conformational orientations were generated, and electrostatic and van der Waals interactions between the ligand and macromolecule were obtained. The result with the lowest binding energy was selected as the best using cluster analysis, visualized using Pymol.

### 2.9. Statistical Analysis

All the experiments were carried out in triplicate, and the results are expressed as mean ± standard deviation (SD). GraphPad Prism 8.0.2 (GraphPad Software Inc., San Diego, CA, USA) was used to perform one-way analysis of variance (ANOVA), and Origin 2021 (Origin Lab Co., Northampton, MA, USA) was applied for mapping. In this study, *p*-values less than 0.05 were considered significantly different.

## 3. Results and Discussion

### 3.1. Effects of Linalool on Cell Morphology of P. fragi

**Figure 1:**
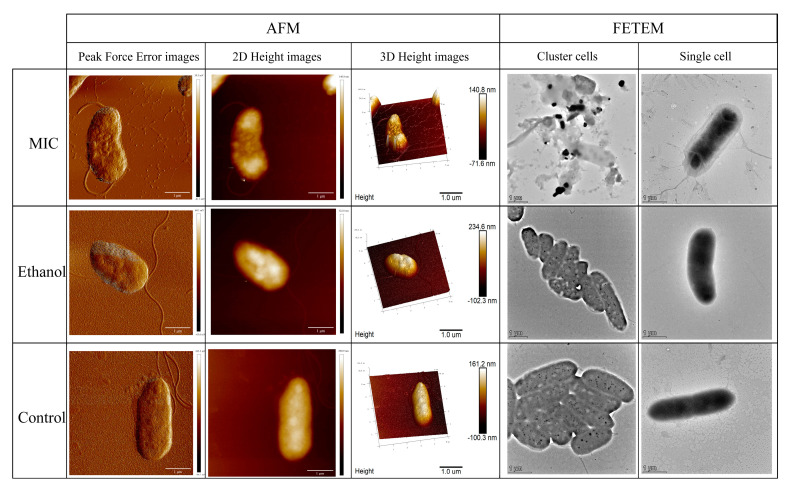
AFM images and FETEM micrographs of *P. fragi* treated with or without linalool for 4 h. MIC: cells treated with 1.5 mL/L of linalool. Ethanol: cells treated with ethanol. Control: cells treated with water.

The cell membrane is the essential structure for maintaining normal function; it is also the first line of defense against external invasion. In this study, the AFM and FETEM observation demonstrated the disruptive effects of linalool on the cell membrane of *P. fragi*. AFM is used extensively for observing bacterial surface details and cell integrity because of its high resolution and 3D imaging capability [38]. The AFM peak force error images and height (2D, 3D) images of *P. fragi* are shown in Figure 1. Peak force error images indicated that the cell surface collapsed with the treatment of linalool. As evidenced from height images, the ethanol and control cells presented a typical rod shape and smooth surface, while the cell surface of the treated group was wrinkled and rough. FETEM is also commonly used to observe structural changes in cell membranes and organelles after exposure to antibacterial agents [39]. Therefore, the FETEM was also used to observe the microstructure of *P. fragi* in this study. Without linalool, the appearance and structure of *P. fragi* were full and complete. However, linalool-treated cells were clearly disturbed, even ruptured and dissolved [40]. By combining AFM with FETEM observation, we could clearly detect the morphological changes in *P. fragi* cells treated with linalool for 4 h. The cell surface in the treatment group was collapsed and swelled, with obvious cell debris and complete separation of membrane structure. As noted by other previous research, such morphological changes could also be observed when different types of bacteria were treated with linalool [41]. A logical explanation is that linalool treatment causes cell membrane breakdown and the leakage of cellular substances, resulting in distortion of bacterial morphology [42].

### 3.2. Effects of Linalool on Cell Membrane Integrity of P. fragi

**Figure 2:**
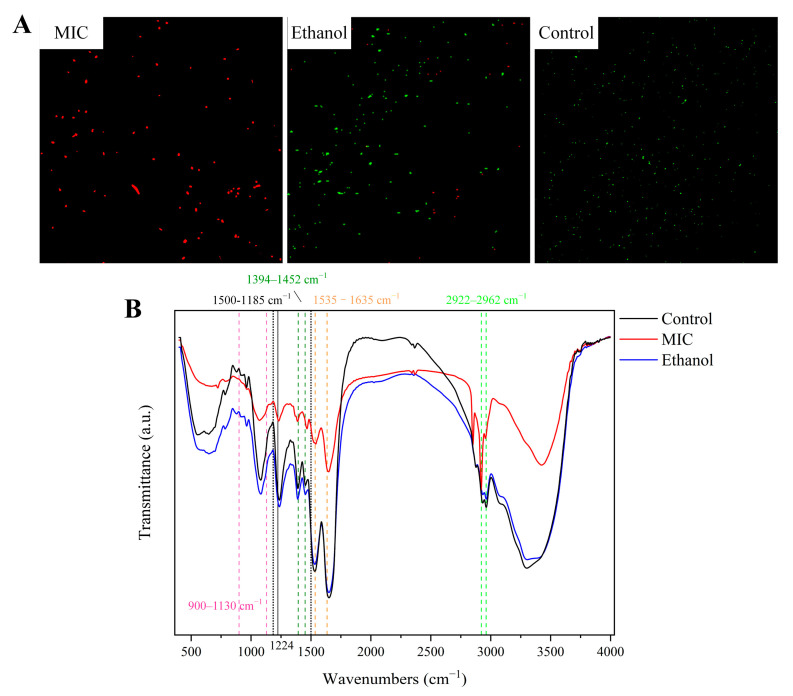
Calcein-AM/PI dual-stained confocal laser scanning microscopy (**A**). Representative FTIR spectra (4000–400 cm^−1^) of *P. fragi* (**B**). MIC: cells treated with 1.5 mL/L of linalool. Ethanol: cells treated with ethanol. Control: cells treated with water.

Based on the alteration of membrane morphology and structure, the cell membrane may be one of the inhibitory targets of linalool, meaning that the integrity of the cell membrane may be disrupted. The cell membrane integrity of *P. fragi* was next evaluated using CLSM combined with fluorescent probes (Calcein AM/PI). Calcein-AM combined with PI can stain and label living and dead cells. As shown in Figure 2A, the control cells were almost green, reflecting that cell membranes were intact. However, *P. fragi* treated with the MIC of linalool emitted strong red fluorescence, indicating that cell membranes were disrupted. The difference in fluorescence revealed that linalool can disrupt the cell membrane, thereby allowing the PI probe to bind to DNA components. FTIR spectroscopic analysis can reflect the stretching and bending vibrations of molecular bonds or functional groups in lipids, protein, DNA/RNA and carbohydrates [29]. The FTIR results highlighted the changes in the intracellular microenvironment, membrane composition, and cytoarchitecture. The cell membrane is an important barrier of the cell intracellular microenvironment and an important part of the cytoarchitecture. In this study, the FTIR signatures between ethanol and the control group were similar, whereas the linalool-treated group was different. It was previously shown that the cell structure damage and membrane leakage increased with the increasing linalool concentration when *Shewanella putrefaciens* was treated with a 0.5 MIC, MIC and 2 MIC of linalool, respectively [43]. The differences in peak values in FTIR spectra (Figure 2B) observed at 2962 and 2922 cm^−1^ (alkyl groups of lipids) and phosphodiester at 1224 cm^−1^ (P=O asymmetric stretching) may be related to the destruction of the cell membrane structure under linalool stress. Because lipids were abundant in cell membranes, it made sense that their bands changed under linalool stress [44]. It is well known that a band at 1700–1500 cm^−1^ can be employed to detect protein backbone conformations. In regard to linalool-treated samples, the changes in transmittance corresponding to 1635 and 1535 cm^−1^ (β sheets of amide I and N-H bonds of amide II) and 1452 and 1394 cm^−1^ (structural protein) revealed that proteins were denatured and modified due to cell lysis and loss of content. In DNA/RNA, the shift in peak values at approximately 1500–1185 cm^−1^ was mainly because of the vibrational contributions. This change in spectra may be due to the leakage of cell intracellular nucleic acids. The increase in intensity observed in the 900–1130 cm^−1^ band showed that new polysaccharides and oligosaccharides were produced. We previously found that linalool can cause energy depletion and structural damage, so the production of polysaccharides and oligosaccharides may be an attempt by bacteria to remodel damaged cellular structures and emergency energy supply [45]. As previously reported, under emergency conditions, *Pseudomonas aeruginosa* upregulates substances for the energy production pathway to prevent energy consumption and carbon leakage [46]. Based on the FTIR results, changes in protein conformation as well as lipid and carbohydrate contents lead to changes in membrane permeability and integrity, resulting in DNA leakage. The cell substances leaked, and the morphology of *P. fragi* changed from rod-shaped cells to amorphous cells, which is consistent with our electron microscopic observations.

### 3.3. Effects of Linalool on Intracellular Proteins and Nucleic of P. fragi

**Figure 3:**
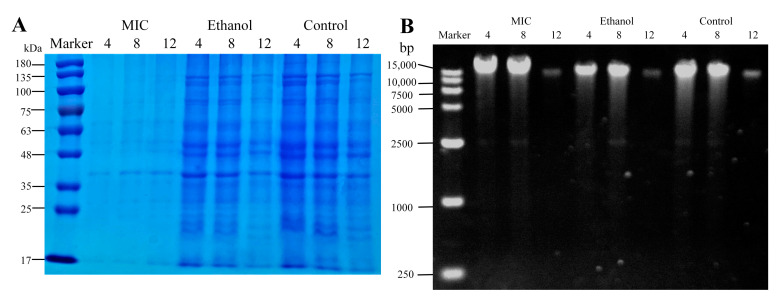
SDS-PAGE analysis of intracellular soluble proteins of *P. fragi* (**A**). DNA release results based on gel electrophoresis in *P. fragi* (**B**). MIC: cells treated with 1.5 mL/L of linalool. Ethanol: cells treated with ethanol. Control: cells treated with water.

Proteins perform important functions within cells and can maintain the normal functioning of organisms. SDS-PAGE analysis was used to identify differences between treated and control groups from protein profiles. The protein bands of markers and samples are shown in Figure 3A. Each band in the marker represented the size of proteins ranging from 17 kDa to 180 kDa. As shown in Figure 3A, the protein bands of the MIC group were dimmed and blurred, while bands of untreated *P. fragi* were clear and clustered. The result indicated that intracellular protein levels were significantly decreased under linalool treatment. The loss of soluble proteins reflected that the linalool had an important effect on *P. fragi*. One possible explanation was that linalool disrupted the cell membrane, resulting in the release of cell contents. The other was that linalool affected protein synthesis in *P. fragi*, and even inhibited the synthesis of enzymes and expression of DNA [47]. Subsequently, the changes in nucleic acid content in *P. fragi* were further measured; the result is shown in Figure 3B. No significant changes in the brightness of gel electrophoresis bands in the MIC and control groups were found, suggesting that linalool treatment had no significant effect on DNA molecular weight, so we proceeded to examine other aspects of the mechanism. Previous proteomics research found that the effects of bacteriostatic agents on DNA may occur by inhibiting RNA processing, transport, and translation, thus accelerating the decline in protein levels in vivo [48], consistent with the results of SDS-PAGE.

### 3.4. Effects of Enzyme Activity Related to Respiration and Energy

**Figure 4:**
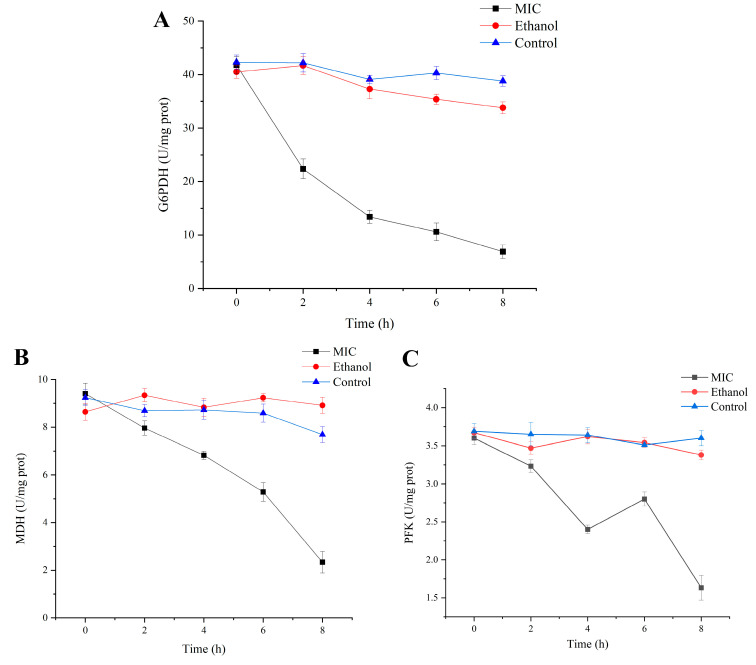
Inhibitory effect of linalool on the enzymatic activity: G6DPH activity (**A**), MDH activity (**B**), PFK activity (**C**). MIC: cells treated with 1.5 mL/L of linalool. Ethanol: cells treated with ethanol. Control: cells treated with water.

Bacteria have a fascinating energy metabolism, which enables them to survive in a wide variety of harsh surroundings [49]. Thus, enzymes in the energy-producing pathway are often targets for antimicrobial activity when bacteria are under environmental stress. To understand the inhibitory effect of linalool on respiratory metabolism, the activities of three key enzymes in energy and respiratory metabolism were determined. It has been proved that the hexose monophosphate (HMP) pathway can phosphorylate glucose to glucose-6-phosphate (G6P) in *Pseudomonas* spp. [50]. Subsequently, G6PDH converts G6P to glucose-6-phosphoate acid, and then to ribose-5-phosphate, which are components of nucleic acids and nucleotides. As shown in Figure 4A, the activity of G6PDH in untreated cells was relatively stable with a slight decrease, while in linalool-treated group it decreased from 41.81 ± 1.58 U/mg prot to 6.97 ± 1.26 U/mg prot. The decrease in G6PDH enzyme activity affected the rate-limiting reaction in HMP pathway and nucleic acid synthesis. Previous studies have found that citronellal reduces the activity of *Escherichia coli* G6PDH, and molecular docking results suggest that citronellal interacts with amino acids in the G6PDH active site [51]. In addition, it has also confirmed that chitosan can cause the leakage of G6PDH in *Pseudomonas aeruginosa* and *Staphylococcus aureus*, which may also represent one of the important reasons for the reduction in G6PDH activity [52].

MDH is a key enzyme involved in the tricarboxylic acid (TCA) cycle, catalyzing the reversible oxidation of malate to oxaloacetic acid. In addition, MDH also plays a necessary role in the malate-aspartate shuttle system and fatty acid oxidation [53]. Therefore, MDH activity was determined in order to represent the level of cellular metabolism [54]. The results (Figure 4B) showed that linalool treatment resulted in the loss of MDH activity, which may be a key component of its antibacterial mechanism.

The first step in Embden–Meyerhof–Parnas (EMP) pathway is catalyzed by PFK, which converts fructose-6-phosphate to fructose-1,6-bisphosphate in an irreversible reaction. PFK is one of the key rate-limiting steps and plays a vital role in the regulation and flux of EMP pathway. In addition, the HMP pathway also depends on the activity of PFK; thus, the activity of PFK was also evaluated [55]. As shown in Figure 4C, the activity of PFK fluctuated over time and eventually decreased from 3.62 ± 0.082 U/mg prot to 1.63 ± 0.16 U/mg prot, demonstrating that the EMP pathway was affected by linalool. Under linalool treatment, *P. fragi* will conserve energy by reducing its own energy metabolism; thus, key enzyme activities of the HMP pathway, TCA cycle, and EMP pathway were reduced. A previous report also confirmed that linalool has a negative effect on enzyme activities involved in the energy production pathway [43]. This may be due to direct damage to the energy metabolism system by linalool, or a derivative effect of increased membrane permeability and amino acid limitation induced by linalool [56].

### 3.5. Analysis of Amino Acid Metabolism

**Figure 5:**
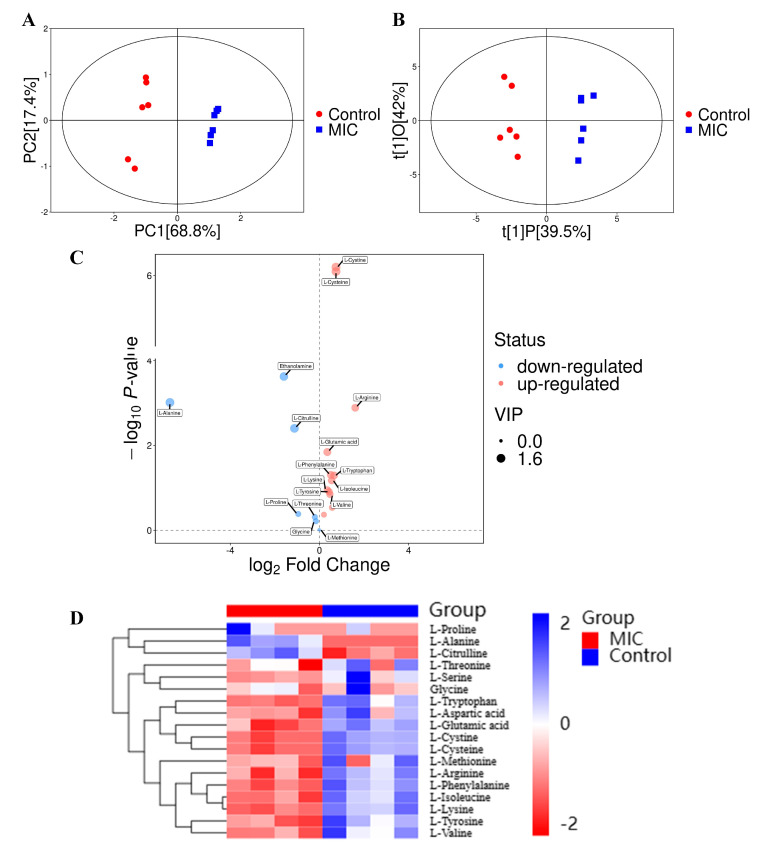
Score plots of PCA (**A**) and OPLS-DA (**B**). Volcano plot (**C**). Upregulated, downregulated and non-significant differential metabolites are represented by red, blue, grey dots, respectively. Heat map (**D**).

Amino acids are one of the substances necessary for the biological process of bacteria. They are involved in protein synthesis, biological enzyme synthesis, gene expression and other important life processes. It has been reported that in times of low energy supply, cells are used to maintain survival by activating amino acids and utilizing specific metabolic pathways [57]. Therefore, in this experiment, the intracellular amino acid profile of *P. fragi* under linalool treatment was determined using UHPLC-MS. Principal component analysis (PCA) and orthogonal partial least squares discriminant analysis (OPLS-DA) revealed the differences in amino acids between the linalool-treated and untreated groups. As shown in Figure 5A, the distribution of scatter points corresponding to the samples of *P. fragi* treated with the MIC of linalool was relatively clustered, indicating good intra-group reproducibility. Meanwhile, the distribution of the two groups was well differentiated on the first principal component (PC [1]). The above results indicated significant changes in the level of amino metabolism of *P. fragi* treated with linalool. According to the OPLS-DA (Figure 5B), the samples of two groups clustered in different regions, suggesting that the model based on UHPLC-MS analysis was able to distinguish the amino acids in *P. fragi* under different treatments. 

The metabolites were statistically analyzed between the MIC group and control group according to the screening criteria of VIP > 0.5 and *p* < 0.5. As shown in the volcano plot (Figure 5C), 16 amino acids were differentially expressed, with 6 amino acids downregulated and 10 amino acids upregulated. In order to visualize the difference between the two groups, the amino acids data have been presented in a heat map. As can be seen in Figure 5D, there were differences in the metabolic levels of various amino acids such as cysteine, glutamic acid and citrulline, which were represented by the high degree of variability in the color blocks. 

**Figure 6:**
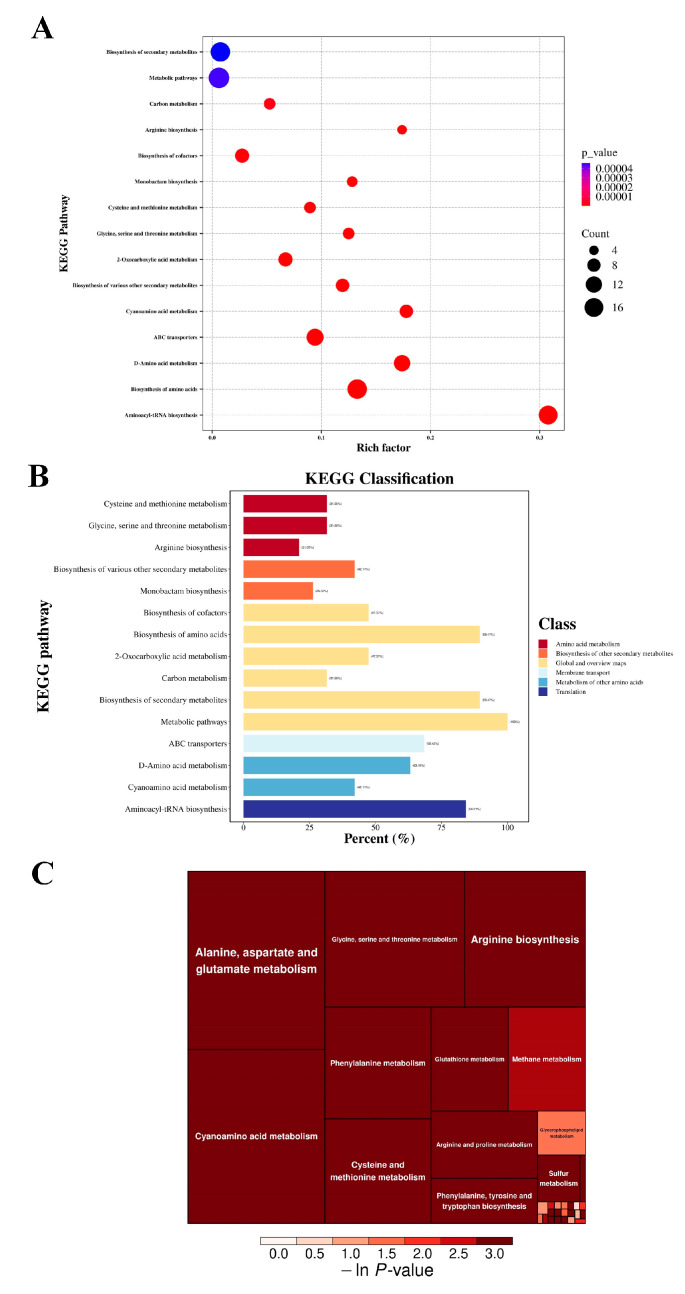
Bubble plot (**A**); each bubble represents a metabolic pathway. KEGG pathway classification (**B**). Enrichment analysis of the amino acids present as a tree map (**C**).

KEGG analysis was applied to identify the metabolic pathways involving differential amino acids. As shown in the bubble plot (Figure 6A), the pathways most affected by differential amino acids through the KEGG pathway enrichment analysis were the metabolic pathway, biosynthesis of secondary metabolites, biosynthesis of amino acids and aminoacyl-tRNA biosynthesis of *P. fragi*. Amino acid metabolism and protein translation are linked by the reaction of aminoacyl-tRNA synthesis. Amino acids bind to tRNA via aminoacyl-tRNA synthetase, which transports the amino acids to the nascent peptide chain for protein synthesis. Therefore, when amino acid metabolism was disturbed in *P. fragi* under linalool stress, aminoacyl-tRNA synthesis was affected accordingly. In the classification of the above pathways (Figure 6B), the representative KEGG functional categories were amino acid metabolism and the metabolism of other amino acids. Refinement of the pathways contained in amino acid metabolism and the metabolism of other amino acid subclasses are presented as a tree map (Figure 6C). The results revealed that the data were highly enriched and possibly related to amino acid mainly concentrated in alanine, aspartate and glutamate metabolism; cyanoamino acid metabolism; glycine, serine and threonine metabolism; arginine biosynthesis; cysteine and methionine metabolism; and phenylalanine metabolism.

Metabolites generated from alanine can be used to synthesize peptidoglycan and teichoic acid, which support and protect the cell structure [58]. In this regard, the decrease in alanine confirmed the indirect disruptive effect of linalool on cell membranes, consistent with the morphological observations [59]. Changes in arginine and proline affected the intracellular ROS levels in *P. fragi* and were shown to be important amino acids in protecting the structural integrity of cell membranes [60]. Glutamic acid and glycine can be converted to glutathione; thus, their upregulation improved the total antioxidant capacity of bacteria. Moreover, glutamate plays an important role in maintaining homeostasis of amino sugar, nucleotide sugar metabolism and peptidoglycan biosynthesis.

**Figure 7:**
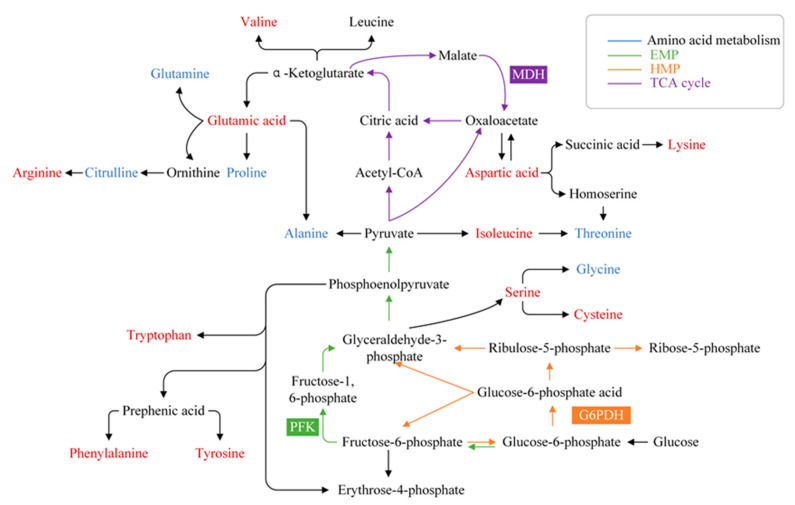
Pathway analysis of enzymes and amino acids related to amino acid metabolism, EMP (green arrows), HMP (orange arrows) and TCA cycle (purple arrows) in *P. fragi* with linalool treatment; red color represents upregulation of amino acids and blue color represents downregulation of amino acids.

As shown in Figure 7, it can be seen that amino acid metabolism also affects energy metabolism such as the TCA cycle, HMP and EMP pathway. When bacteria were stressed by the linalool, the decrease in threonine and alanine was due to pyruvate production, which supplied energy to bacteria. Glyceraldehyde-3-phosphate is an important intermediate in the HMP and EMP pathway and can control serine synthesis. Subsequently, the upregulation of serine affected glycine and cysteine synthesis. Glutamic acid can be converted to α-ketoglutaric acid, and the upregulation of its content suggests that it provided energy support for bacteria under stress in the TCA cycle [61]. Oxaloacetate can be used as a carbon skeleton of amino acids to synthesize aspartic acid [62]. When aspartic acid was upregulated, the production of oxaloacetate can be inhibited, resulting in reduced MDH activity. Therefore, when the synthesis of amino acids in cells was disturbed, the energy metabolism was also affected. The reason for this phenomenon may be that in the presence of linalool, *P. fragi* used amino acids as an emergency energy supply, while blocked energy production pathways lead to slow amino acid synthesis, which is consistent with previous studies [63].

### 3.6. qRT-PCR

**Figure 8:**
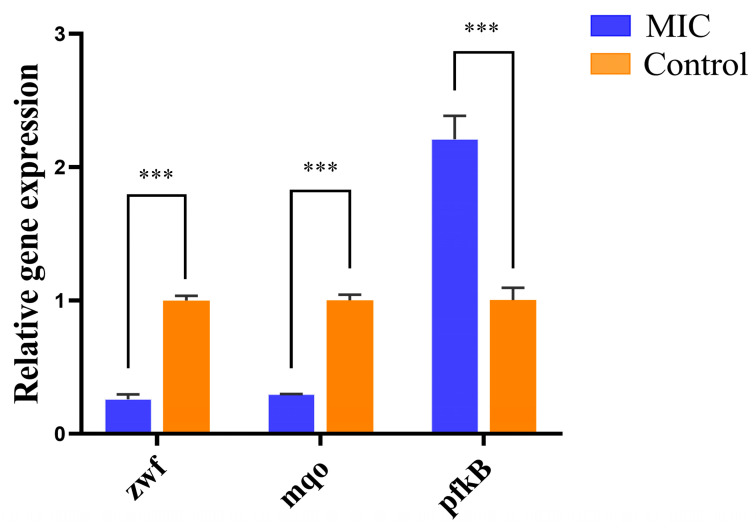
Validation of selected DEGs using real-time PCR. *** *p* < 0.001.

To understand the mechanism of linalool reducing enzyme activities in TCA cycle, HMP and EMP pathway, the gene expressions of *zwf*, *mqo* and *pfkB* were examined. As shown in Figure 8, linalool has a significant effect on the expression of genes in *P. fragi*. The *zwf* gene encodes G6PDH and plays an essential role in many reactions. It was discovered that the absence of *zwf* would prevent G6P from entering the HMP pathway via G6PDH in mutants [64]. The result in Figure 8 shows that the expression of *zwf* was reduced to 26%, possibly leading to a decrease in the activity of G6PDH. Malate quinone oxidoreductase (MQO) is encoded by *mqo*, which is a lipid-dependent peripheral membrane protein that also takes part in the TCA cycle. It catalyzes the formation of oxaloacetate from L-malate and participates in the electron transport chain [65]. Higher TCA cycle flux can be obtained with MQO compared to MDH because it does not depend on the NADH/NAD and malate/oxaloacetate ratios [66]. Thus, MQO is essential for maintaining stable TCA cycle fluxes under favorable conditions. The results demonstrate that linalool significantly downregulated gene expression of *mqo* to 30%. The key enzymes in EMP are PFK I and II, encoded by *pfkA* and *pfkB*, respectively. The synthesis of fructose-1,6-bisphosphatase catalyzed by *pfkB* is not regulated by substrates and metabolites. Therefore, when *pfkA* function is inhibited, EMP pathway can be carried out normally with *pfkB*. Thus, when most transcripts encoding EMP enzymes were downregulated, the expression of *pfkB* was significantly upregulated under stress conditions [67]. Consistent with our result, the expression of *pfkB* was upregulated by a factor of 2.209 compared to the control group.

### 3.7. Molecular Docking

**Figure 9:**
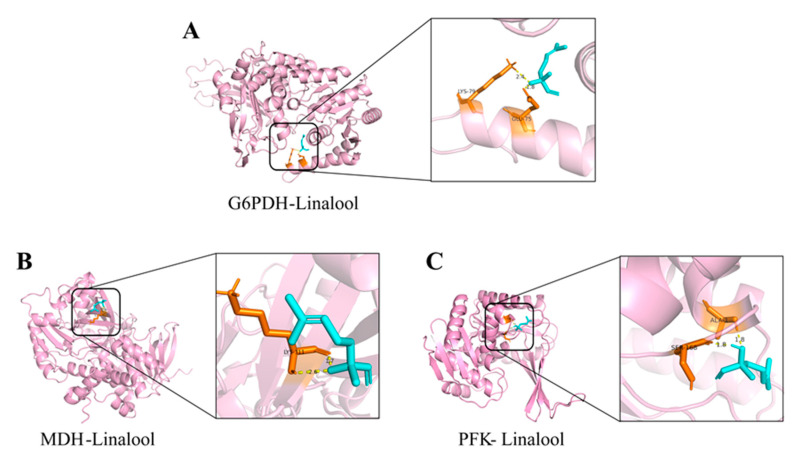
Molecular docking perspective of linalool with the binding sites of G6PDH (**A**), MDH (**B**) and PFK (**C**). Ligand: green color, macromolecule: pink color, receptor: orange color.

Molecular docking could reveal a potential interaction between the ligand and macromolecules. In this study, linalool was used as the only ligand to dock with enzymes (G6PDH, MDH, and PFK). From the docking model, linalool was inserted into cavities on the protein surfaces and bound to the amino acid residues [68]. The lowest docking energy of G6PDH and linalool was −3.54 kcal mol^−1^. The H18 of linalool formed hydrogen bonds with Glu-75 and Lys-79 of G6PDH with bond lengths of 1.8 Å and 2.4 Å, respectively (Figure 9A). Linalool was predicted to make contact with the MDH model (−3.07 kcal mol^−1^), forming hydrogen bond with Lys-311 amino acid residue (Figure 9B). The results shown in Figure 9C indicate that linalool formed two hydrogen bonds with Ser-168 and Ala-171 amino acid residues of PFK (−3.38 kcal mol^−1^). Linalool was mainly bound to these enzymes through hydrogen bonding. One possible explanation for this is that linalool may compete with small molecules, thereby blocking substrate binding at the active site [69]. Another hypothesis is that the combination of linalool and amino acid at the active site caused changes in the nearby spatial structure. The structure of proteins will affect the function, thereby altering the activity of enzymes. As a consequence, the interaction of residues with linalool may contribute to the inhibition of the key step in the physiological metabolism.

## 4. Conclusions

In summary, this study combines, for the first time, targeted amino acid metabolomics and a molecular approach to gain an insight into the antibacterial mechanism of linalool against foodborne *P. fragi*. This study’s results showed that linalool exhibited effective inhibition of *P. fragi* with an MIC of 1.5 mL/L. Linalool treatment resulted in altered cell morphology of *P. fragi*, accompanied by disruption of the cell membrane structure leading to leakage of intracellular proteins. In addition, the disruption of the cell membrane caused an imbalance in the homeostasis of the intracellular environment, and the amino acid and energy metabolism of *P. fragi* was disturbed, resulting in the interruption of life activities and even death of the bacteria. Briefly, the anti-*P. fragi* mechanism of linalool was summarized as cell membrane structure disruption (leakage of intracellular proteins), amino acid metabolism limitation (affected the catabolism and synthesis of cysteine, glutamic acid and citrulline) and energy metabolism disorder (inhibition of the gene expression levels and activities of G6PDH, MDH, and PFK). These findings highlight the great promise of developing linalool against *P. fragi* and provide new ideas for using linalool as a natural antibacterial agent.

## Figures and Tables

**Table 1:** Primer sequences for qRT-PCR.

Gene Name	Nucleotide Sequence (5′→3′)	Direction Primer	Location (nt)	Product Length (bp)
*16s RNA*	CGGGAACATTGAGACAGG	Forward	1006	121
	AGAGTGCCCACCATTACG	Reverse	1126	
*zwf*	GCTGCTGTGCCTGATTGC GTCGCTCTGGGTGTTGGA	Forward Reverse	720 930	211
*mqo*	AAAGACGGCAGCACAACC ACGGGCCAAACAGGATGA	Forward Reverse	745 1021	277
*pfkB*	GCAGGGTGTCGAGCATGTAGT CGCTGAGCAAACCGTGGA	Forward Reverse	639 799	161

## Data Availability

The original contributions presented in this study are included in the article; further inquiries can be directed to the corresponding author.
